# The Importance of Natural Antioxidants in the Treatment of Spinal Cord Injury in Animal Models: An Overview

**DOI:** 10.1155/2019/3642491

**Published:** 2019-11-12

**Authors:** Angélica Coyoy-Salgado, Julia J. Segura-Uribe, Christian Guerra-Araiza, Sandra Orozco-Suárez, Hermelinda Salgado-Ceballos, Iris A. Feria-Romero, Juan Manuel Gallardo, Carlos E. Orozco-Barrios

**Affiliations:** ^1^CONACyT-Unidad de Investigación Médica en Enfermedades Neurológicas, Hospital de Especialidades Dr. Bernardo Sepúlveda, Centro Médico Nacional Siglo XXI, Instituto Mexicano del Seguro Social, Mexico City, Mexico; ^2^Unidad de Investigación Médica en Enfermedades Neurológicas, Hospital de Especialidades Dr. Bernardo Sepúlveda, Centro Médico Nacional Siglo XXI, Instituto Mexicano del Seguro Social, Mexico City, Mexico; ^3^Unidad de Investigación Médica en Farmacología, Hospital de Especialidades Dr. Bernardo Sepúlveda, Centro Médico Nacional Siglo XXI, Instituto Mexicano del Seguro Social, Mexico City, Mexico; ^4^Unidad de Investigación Médica en Enfermedades Nefrológicas, Hospital de Especialidades Dr. Bernardo Sepúlveda, Centro Médico Nacional Siglo XXI, Instituto Mexicano del Seguro Social, Mexico City, Mexico

## Abstract

Patients with spinal cord injury (SCI) face devastating health, social, and financial consequences, as well as their families and caregivers. Reducing the levels of reactive oxygen species (ROS) and oxidative stress are essential strategies for SCI treatment. Some compounds from traditional medicine could be useful to decrease ROS generated after SCI. This review is aimed at highlighting the importance of some natural compounds with antioxidant capacity used in traditional medicine to treat traumatic SCI. An electronic search of published articles describing animal models of SCI treated with natural compounds from traditional medicine was conducted using the following terms: Spinal Cord Injuries (MeSH terms) AND Models, Animal (MeSH terms) AND [Reactive Oxygen Species (MeSH terms) AND/OR Oxidative Stress (MeSH term)] AND Medicine, Traditional (MeSH terms). Articles reported from 2010 to 2018 were included. The results were further screened by title and abstract for studies performed in rats, mice, and nonhuman primates. The effects of these natural compounds are discussed, including their antioxidant, anti-inflammatory, and antiapoptotic properties. Moreover, the antioxidant properties of natural compounds were emphasized since oxidative stress has a fundamental role in the generation and progression of several pathologies of the nervous system. The use of these compounds diminishes toxic effects due to their high antioxidant capacity. These compounds have been tested in animal models with promising results; however, no clinical studies have been conducted in humans. Further research of these natural compounds is crucial to a better understanding of their effects in patients with SCI.

## 1. Introduction

Spinal cord injury (SCI) is a life-disrupting condition associated with high mortality and long-term morbidity, which may provoke severe consequences to patients, such as paraplegia or quadriplegia, and frequently continues as a terminal condition. According to the National SCI Statistical Center, an annual incidence of 17,500 new SCI cases is estimated. Since it is a frequent and severe motor injury, it becomes a potential economic, social, and family burden. In 2017, between 245,000 and 353,000 patients with SCI were alive in the United States, with an estimated lifetime cost of $1.6-$4.8M per patient [1].

Among the leading causes of SCI are traffic and sports accidents, as well as falls and violence. Traumatic SCI occurrence shows a peak between the ages of 15 and 29 years and another peak over the age of 65 years, with an incidence rate of 3–4 times higher in males [2, 3]. Depending on the severity of the lesion, patients show neurological deficits, which can range from loss of sensation to death, including paralysis, impaired bowel, bladder, and sexual function, as well as autonomic dysfunction [4–7].

Natural antioxidants are used as an alternative treatment for some neuropathologies, including SCI. This review is aimed at providing an overview of various natural compounds that produce beneficial effects for the treatment of SCI in animal models. Furthermore, the differences between animal responses to these various compounds are addressed to establish a better understanding of the cellular and molecular mechanisms occurring in the spinal cord following injury.

## 2. Pathophysiology of SCI

SCI can be divided into primary, secondary, and chronic phases [8, 9]. The first phase is the result of the physical forces involved in the initial traumatic event, which commonly are the most critical elements of injury severity. These forces include compression, shearing, laceration, and acute stretch/distraction [10]. After the initial injury, a cascade of subsequent events is initiated. In this second phase, the injury expands and neurological deficits and outcomes are exacerbated [11, 12]. Secondary SCI is a delayed and progressive injury following the first damage. Finally, a chronic phase, begins days to years after the injury, leading to neurological impairments in both orthograde and retrograde directions, including some brain regions [13, 14].

During the secondary cascade, some vascular changes are observed [15]. Furthermore, neutrophils and macrophages release superoxide anion and hydrogen peroxide as a means to sterilize the injury site. Infiltrating activated hematogenous phagocytic cells and tissue macrophages generate massive quantities of superoxide anion by nicotinamide adenine dinucleotide phosphate (NADPH) oxidase as its primary source [16].

In addition, phagocytic inflammatory cells release reactive oxygen species (ROS). Free radicals react with polyunsaturated fatty acids leading to peroxidation and disruption of the typical phospholipid architecture of cellular and subcellular organelle membranes. Moreover, lipid peroxidation generates aldehyde products that impair the function of key metabolic enzymes, such as Na^+^/K^+^-ATPase [17].

SCI is generally characterized by an increase in cytokines, such as TNF-*α*, IL-1*β*, and IL-6 that lead to upregulation of inflammatory and apoptotic agents, including NF-*κ*B, AP-1, JNK, p38 MAPK, and PGE2 [3].

After SCI, an upregulated liberation of excitatory amino acids, such as glutamate and aspartate, is observed due to the release from disrupted cells [18–20].

Finally, in the chronic phase, the glial scar, which is integrated by reactive astrocytes, microglia/macrophages, and extracellular matrix molecules—chondroitin sulfate proteoglycans in particular—prevents axon growth through it by acting as a physical barrier [21–25].

Consequently, it becomes necessary to develop reliable strategies and treatments for SCI patients. An essential strategy for SCI treatment is the reduction of ROS levels, which could be carried out using antioxidants or compounds that regulate ROS or modify their signaling pathways [26, 27].

## 3. ROS Production and Spinal Cord Injury

### 3.1. Oxidative and Nitrosative Stress

The homeostasis of redox mechanisms in the spinal cord is maintained in balance. However, under adverse conditions such as neurodegenerative diseases or traumas, this balance is altered. The group of biochemical and molecular reactions following SCI is called secondary damage. The most extensively studied and accepted mechanism of secondary damage is the injury produced by oxidative and nitrosative stress [28].

**Figure 1:**
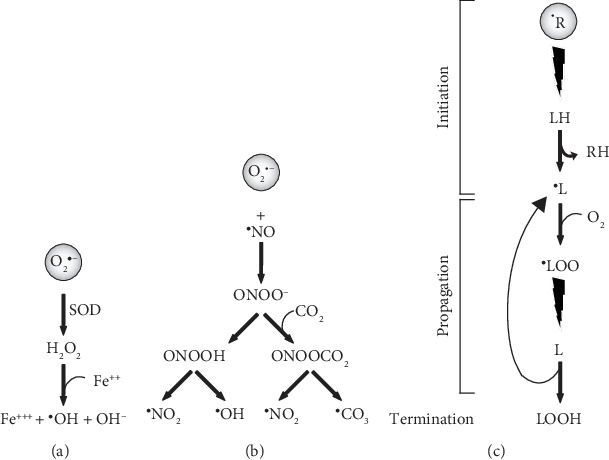
Main pathways of ROS and NOS in the central nervous system. (a) Fenton reaction; (b) peroxynitrite reaction; (c) lipid peroxidation. O_2_^·^-: superoxide radical; SOD: superoxide dismutase; H_2_O_2_: hydrogen peroxide; Fe^++^: ferrous iron; Fe^+++^: ferric iron; ^·^OH: hydroxyl radical; OH-: hydroxyl anion; ^·^NO: nitric oxide or nitrogen monoxide; ^·^NO_2_: nitrogen dioxide; ONOO-: peroxynitrite anion; CO_2_: carbon dioxide; ONOOH: peroxynitrous acid; ONOOCO_2_: nitrosoperoxocarbonate; ^·^CO_3_: carbonate radical; ^·^R: free radical; RH: neutralized radical; LH: polyunsaturated fatty acid; ^·^L: alkyl radical; O_2_: molecular oxygen; ^·^LOO: peroxyl radical; LOOH: lipid hydroperoxide.

The essential molecule within oxidative stress is the superoxide (O_2_^·-^) radical, which is produced by the reduction of an electron in an O_2_ molecule. This radical has ambivalent functions: it can act as an oxidizing or reducing agent. Despite being considered a modestly reactive free radical, it can react with other molecules to generate more reactive free radicals (Figure 1(a)). For example, the nitric oxide (^·^NO) radicals produce peroxynitrite (ONOO^−^), which has a higher oxidation potencial. Consecutively, the ONOO^−^ radical can be protonated to form peroxynitrous acid (ONOOH), which in turn can be decomposed into two highly reactive molecules, nitrogen oxide and hydroxyl radical (^·^NO_2_ and ^·^OH). Furthermore, the ONOO^−^ radical interacts with carbon dioxide (CO_2_) as well, to produce nitrosoperoxycarbonate (ONOOCO_2_), which decomposes into nitrogen oxide and carbonate (^·^CO_3_) radical (Figure 1(b)) [29].

Moreover, O_2_^·-^ can be dismuted by the superoxide dismutase (SOD) enzyme to form hydrogen peroxide (H_2_O_2_) in the presence of Fe^2+^, which is later oxidized to Fe^3+^, ^·^OH and OH^−^ (Fenton reaction). Fe^3+^ is released from its transporter and storage proteins (transferrin and ferritin, respectively) by pH acidification due to the traumatic impact (Figure 1(a)). Additionally, Fe is also released by the hemoglobin resulting from trauma [30].

### 3.2. Lipid Peroxidation

Lipid peroxidation (LP) is the oxidative degradation of unsaturated fatty acids, such as arachidonic acid, linoleic acid, eicosapentaenoic acid, and docosahexaenoic acid, by the action of oxygen-free radicals, which causes disruptions in the integrity of the cell membrane [31].

This process is carried out in three stages: initiation, propagation, and termination. The initiation phase occurs when a free radical (^·^R) attacks and removes hydrogen along with its single electron from an allylic carbon of the fatty acid (LH), generating an alkyl radical (^·^L). The propagation phase starts with the participation of O_2_ to form the peroxyl radical (^·^LOO). Subsequently, the radical ^·^LOO reacts with another fatty acid converting it into another alkyl radical ^·^L and LOOH, propagating the oxidative state in a series of chain reactions leading to the destabilization of the membrane (Figure 1(c)). These reactions are terminated by the depletion of substrates, encountering another radical or a scavenger and ending with nonradical molecules. Within this group of reactions, a couple of toxic aldehyde products, 4-hydroxynonenal (4-HNE) and 2-propenal (acrolein), are generated. The toxicity of 4-HNE and acrolein lies in their ability to bind to protein amino acids, altering their structure and function.

In contrast, another nontoxic product, malondialdehyde (MDA), together with the quantification of 4-HNE and acrolein, is widely used to measure LP levels. These markers have allowed the characterization of the LP in SCI models [32–34].

LP is an event that appears within the first 30 minutes after SCI and increases considerably from the first hour to a maximum point three hours after the SCI in a contusion model [35].

Another type of damage is caused by the ^·^NO radical which, as mentioned above, reacts with the radical O_2_^·-^ producing the ONOO^−^ radical. This radical produces the nitration of proteins when interacting with the amino acid tyrosine, generating posttranslational modifications by converting tyrosine into 3-nitrotyrosine (3-NT). 3-NT is used as a biological marker of the action of ONOO^−^ [36] and has been detected within the first hour after the injury and for several days. Nitric oxide synthase (NOS) in all its isoforms, including a mitochondrial variant, is responsible for the production of the radical ^·^NO. The expression of nNOS increases in the CNS after a traumatic injury [37].

## 4. Effects of Natural Antioxidant Compounds on SCI

Medicinal plants have been used for thousands of years. Herbs, roots, bulbs, and fruits contain different compounds which act as therapeutic ingredients [38]. Recently, traditional medicine has been the focus of attention in the treatment of some diseases, including SCI [39].

Due to their antioxidant characteristics and ROS modulation properties, many natural compounds could be useful to reduce ROS generated in the SCI (Table 1). For that reason, it is vital to study the mechanisms of action through which they perform their effects. Therefore, some examples of antioxidant compounds found in several plant species used as SCI treatment are described and discussed as follows.

### 4.1. Extracts from Leaves

#### 4.1.1. Asiatic Acid

Traditional Chinese medicine has offered many proposals, including asiatic acid (AA) and other compounds. AA is extracted from the Chinese herb *Centella asiatica*. It is a pentacyclic triterpenoid compound with anti-inflammatory, hepatoprotective, cardioprotective, neuroprotective, gastroprotective, and anticancer properties [40].

AA was proposed as SCI treatment for its therapeutic potential. Promising results were observed in the model used, in which Sprague-Dawley rats with induced SCI responded to the treatment: scores increased in the tests evaluated, Basso, Beattie, and Bresnahan (BBB) and inclined plane. Also, AA reduced myeloperoxidase activity, as well as interleukin-1*β* (IL-1*β*), interleukin-18 (IL-18), interleukin-6 (IL-6), tumor necrosis factor-*α* (TNF-*α*), ROS, H_2_O_2,_ and MDA levels. Moreover, the activity of superoxide dismutase (SOD) and the content of glutathione (GSH) increased with AA [41].

The mechanism of AA involves the activation of the nuclear factor- (erythroid-derived 2-) like-2 factor (Nrf2), a cytoprotective factor that regulates the expression of genes encoding antioxidant, anti-inflammatory, and detoxifying proteins, as well as heme oxygenase-1 (HO-1), a protein coded by a Nrf2-dependent gene, which degrades toxic heme groups; produces biliverdin, iron ions, and carbon monoxide; and contributes to angiogenesis [42]. The effects of AA can also be attributed to the inhibition of ROS and the nucleotide-binding domain and leucine-rich repeat- (LRR-) containing (NLR) family pyrin domain-containing 3 (NLRP3) inflammasome pathway. The NLRP3 inflammasome is a multiprotein complex that activates caspase-1, leading to the secretion of proinflammatory molecules such as IL-1*β* and IL-18. Under normal conditions, NLRP3 remains autorepressed, but this changes with increasing concentrations of damage-associated and pathogen-associated molecular patterns as well as ROS, which are crucial elements for NLRP3 activation [43].

SCI can lead to secondary acute lung injury (ALI). AA administration significantly attenuates pulmonary permeability index and pulmonary histologic conditions and exhibits a protective effect on SCI-induced ALI by alleviating the inflammatory response through inhibiting NLRP3 inflammasome activation and oxidative stress with the upregulation of Nrf2 protein levels [44]. The use of AA may be a potential efficient therapeutic strategy for the treatment of SCI and SCI-induced ALI [41, 44].

#### 4.1.2. Ligustilide

Ligustilide (3-butylidene-4,5-dihydrophthalide) is the main lipophilic constituent of the *Umbelliferae* family of medicinal plants, including *Radix angelicae sinensis* and *Ligusticum chuanxiong* [45]. As it crosses the blood-brain barrier, ligustilide (LIG) exerts marked neuroprotective effects against several CNS pathologies, including forebrain ischemic injury in mice, permanent forebrain ischemia and focal cerebral ischemia/reperfusion in rats [46–48]. In addition, LIG exhibited a wide range of pharmacologic effects *in vitro* and *in vivo*: cardioprotective, antioxidant, anti-inflammatory, and neuroprotective activities [49]. Xiao et al. demonstrated that LIG promotes functional recovery in rats with SCI by preventing the production of ROS. Treatment with LIG significantly increased BBB scores and reduced the time for recovery of coordination in rats with SCI. Furthermore, LIG suppressed SCI-induced production of ROS, inducible nitric oxide synthase (iNOS), inflammation, and JNK signaling. However, further studies are needed to identify the mechanisms by which LIG regulates neuroprotection and mediates locomotor recovery following SCI [50].

#### 4.1.3. Tetramethylpyrazine

Tetramethylpyrazine (TMP), an alkaloid extracted from the Chinese medicinal herb *Ligusticum wallichii Franchat* (chuanxiong), is widely used in the treatment of ischemic stroke and cardiovascular disease [51–53] and has shown anti-inflammatory and neuroprotective effects against SCI as well [54, 55]. In different models of SCI, TMP improved locomotor functions when compared with control animals [52, 56–60]. This improvement in motor activity correlated positively with a decreased area of the injury-induced lesion and increased tissue sparing [51, 58, 59]. Furthermore, a decreased permeability of the blood-spinal cord barrier was also observed [55]. TMP promoted angiogenesis increasing vessel number, vessel volume fraction, and connectivity as well [58, 60]. A mechanism proposed for these effects is the overexpression of PGC-1, a transcriptional coactivator linked to energy metabolism in the mitochondria. This protein is involved in a variety of neurological disorders and apoptosis [57]. Additionally, TMP prevents the reduction of HO-1 and Akt phosphorylation produced in SCI [55].

Regarding neuropathic pain produced in SCI, TMP treatment increased both mechanical withdrawal thresholds and thermal withdrawal latencies [53, 61]. The effect of TMP on neuropathic pain relies on neuronal survival in the dorsal horn and the inhibition of astrocyte activation [62]. In the case of neuronal survival, TMP can modulate mediators of apoptosis such as Bcl-2 and caspase-3 [61], whereas, in the case of the inhibition of astrocyte activation, TMP releases matrix metalloproteinase-2/9 (MMP-2/9) to induce central sensitization and maintain neuropathic pain [62]. Moreover, TMP treatment decreased the expression of pSTAT3. Therefore, TMP could attenuate neuropathic pain by the inhibition of the JAK/STAT3 pathway [53].

TMP shows anti-inflammatory effects in SCI: TMP treatment reduced the expression of proinflammatory cytokines TNF-*α*, IL-1*β*, macrophage migration inhibitory factor positive (MIF), IL-18, IL-2, and COX-2 [51–56]; upregulated the expression of anti-inflammatory cytokines IL-10, I-*κ*B, and IL10; inhibited the activation of NF-*κ*B [51, 52]; alleviated neutrophil infiltration; and attenuated microglia activation [51, 52, 54]. Matrix metalloproteinases 2 (MMP2) and 9 (MMP9) are implicated in neuropathic pain by mediating inflammatory pathways. TMP administration induces downregulation of both metalloproteases [60]. Thus, TMP prevents inflammation in spinal cord injury in rats.

TMP reduced neuronal apoptosis by increasing Bcl-2 and reducing Bax, as well as reduced TUNEL-positive cells and caspase-3 and caspase-9 activities [55, 57, 59–61]. Furthermore, TMP increases miR-21 expression, thus decreasing the expression of its targets FasL, PDCD4, and PTEN [59]. In addition to miR-21, TMP decreased the expression of miR-214-3p by increasing the expression of Bcl2L2, suggesting that TMP can modulate apoptosis in SCI [63].

Finally, TMP can decrease ROS in SCI. In rats, TMP treatment decreased lipid peroxidation and increased glutathione levels and superoxide dismutase activity. Also, TMP regulated the expression of Nrf2 mRNA and its binding in HO-1 promoter positively. Thus, TMP showed effects against ROS through the activation of the Akt/Nrf2/HO-1 pathway [55, 56].

#### 4.1.4. Epigallocatechin-3-Gallate

Epigallocatechin-3-gallate (EGCG) is the most abundant polyphenol found in green tea, for which multiple benefits have been described: anticholesterolemic, antioxidant, and anti-inflammatory functions, as well as a modulator of apoptosis. In the CNS, a neuroprotective effect has been shown in a wide range of neurodegenerative diseases in various animal models. In the case of SCI, the administration of EGCG improves both motor and sensory (allodynia, nociception, and hyperalgesia) functions in acute and chronic models. EGCG administration (100 mg/kg weight) produces the recovery of motor function [64–68] accompanied by a decrease of the injury area and an increase in the number of neurons [64]. The main mechanisms underlying the effects of EGCG range from the induction of the expression of neurotrophic factors, such as BDNF, GDNF [66, 69], and NT3, and their receptors, Trk-B, Trk-C, and NGFR-p75 [69], to the expression of growth factors, such as FGF2 and VEGF [70], accompanied by an increase of GAP43 [64], and the attenuation of myelin degradation [65, 66]. *In vitro* models have shown that EGCG decreases the inhibitory activity of neuritic growth and the collapse of the growth cone induced by NOGO-66. This effect was observed through the 67 kDa laminin receptor to which EGCG binds with high affinity [71]. Also, EGCG attenuates axon repulsion mediated by semaphorin [72]. Evidence shows that EGCG has protective effects for the modulation of neurotrophic factors and their receptors, as well as axonal sprouting. EGCG relieves the neuropathic pain produced in SCI and constriction of the sciatic nerve models [67, 68, 73–75], allowing the recovery of sensory functions by improving tactile allodynia and mechanical nociception. EGCG also increases the latency of paw withdrawal and tail-flick tests [67, 68]. Within the molecular mechanisms of EGCG to alleviate neuropathic pain is the reduction of the expression of CX3CL1, a fractalkine chemokine that has been shown to play an essential role in the development of neuropathic pain. The administration of EGCG reduced thermal hyperalgesia, as an effect of the reduction of CX3CL1 protein expression but not its RNA. Therefore, EGCG is suggested to act as a mediator of nociceptive signaling between neurons and glial cells [75].

Another mechanism that has been studied is the suppression of TLR4 expression. Several studies have demonstrated the involvement of TLRs and inflammation in the development of neuropathic pain. Although EGCG can inhibit other effector molecules of inflammation, the sole inhibition of TLR4 can inhibit the TLR4/NF-*κ*B pathway. Also, EGCG induces the decrease of HMGB1, which has been implicated in chronic neuropathic pain by joining to TLR4 and activating the immune response [73].

EGCG has a more significant action on neuropathic pain than on motor recovery. In the short term, the administration of EGCG only affects sensory recovery but not on motor recovery [76].

EGCG can inhibit the expression of RhoA, FASN, and TNF-*α*. In addition to limiting axonal regeneration, Álvarez-Pérez et al. demonstrated that RhoA participates in the generation of neuropathic pain. In the case of FASN, which is an enzyme that synthesizes palmitate, a lipid that is capable of activating the synthesis and release of proinflammatory agents, EGCG induces the decrease of FASN and the activation of the inflammatory pathways involved in neuropathic pain [74].

In addition to the mediators of inflammation described in neuropathic pain and a potent anti-inflammatory effect, EGCG is able to attenuate the activity of myeloperoxidase and attenuate the expression of inflammatory cytokines, such as TNF-*α*, IL-1*β*, IL-6, IL-2, MIP1, RANTES, nitrotyrosine, iNOs, COX, PARP, NF-*κ*B, and HMGB1 [65, 73], accompanied by an increase in the anti-inflammatory cytokine IL-10 as well [73].

EGCG also modified the expression of IL-4, IL-12p70, and TNF-*α*1. The mechanism depends on the nuclear translocation of the p65 subunit of NF-*κ*B, consequently inhibiting its activity along with the inflammatory pathways that it regulates. EGCG can modulate the expression of macrophages type M1 and M2. Both macrophage populations have different actions in the mechanisms of inflammation, allowing opposed states [70]. Therefore, EGCG modulates the expression of inflammatory cytokines that affect the expression and activity of its inductors.

EGCG also has antioxidant effects. In SCI, EGCG significantly reduces MDA levels [64] and increases glutathione reductase. This overexpression is accompanied by the decrease of isoprostanes in urine and the suppression of HO-1 [77].

As survival mechanisms, on the one hand, EGCG increases the expression of Bcl-2 and survivin and, on the other hand, it decreases the expression of Bax [64–66, 68]. These effects are reflected by the TUNEL assay low positivity in treated spinal cords [64]. Furthermore, PARP is a protein that is capable of inducing death by the depletion of NAD and ATP. EGCG decreases the expression of PARP, which could contribute to the reduction of Bax and the increase of Bcl2 [65].

#### 4.1.5. Ginsenosides

Ginsenosides are steroid-like molecules which have a four trans-ring structure with sugar residues attached [78]. Ginsenosides Rb1, Rg1, and Rg3 show multiple pharmacological activities on the cardiovascular and immune systems, as well as neuroprotective effects [79, 80].

Ginsenosides can act as antioxidants or scavengers for free radicals [81], increase the activity of superoxide dismutase, and reduce lipid peroxidation [82, 83].

Rb1 protects neurons against oxidative injury, enhancing the Nrf2/HO-1 pathway since the activation of Nrf2 upregulates the transcription of multiple antioxidant response element-controlled genes [84, 85]. Also, ginsenoside Rb1 protects the ischemic brain through upregulation of the expression of Bcl-xL *in vitro* and *in vivo* [86].

*Panax ginseng* C.A. Meyer (*P. ginseng*) is an herb commonly known as Asian or Korean ginseng [87], which contains ginsenoside saponins that account for the pharmacological efficacy [88].

*P. ginseng* has 150 different types of ginsenoside saponins, but Rb1, Rb2, Rc, Rd, Re, and Rg1 constitute more than 90% of the total ginsenosides [89].

Regarding the effects on SCI, the ginsenosides Rb1 and Rg1, extracted from *Panax ginseng* C.A. Meyer, were efficient neuroprotective agents for spinal cord neurons *in vitro* survival assays. These compounds protected spinal neurons from excitotoxicity induced by glutamate and kainic acid, as well as oxidative stress induced by H_2_O_2_. The neuroprotective effects are dose dependent, which optimal doses were 20-40 mM for both Rb1 and Rg1 [81].

Other authors demonstrated *in vitro* that dgRb1 (dihydroginsenoside Rb1), a stable chemical derivative of gRb1, upregulated VEGF and Bcl-xL expression and facilitated neuronal survival through the hypoxia response element (HRE) and signal transducers and activators of transcription 5 (Stat5) response element [90]. Consistently, Sakanaka et al. showed that an intravenous infusion of dgRb1 improved SCI in rats, as well as ischemic brain damage in MCA-occluded rats. The dgRb1-treated groups showed significant improvement of motor activity and behavioral abnormalities concerning locomotor and rearing activities and BBB score in a dose-dependent manner after SCI [90].

Kim et al. reported that ginseng extracts injected intraperitoneally improved recovery after contusive SCI in rats [91]. Additionally, Zhu et al. showed that the oral administration of red ginseng extract promoted the recovery from the motor and behavioral abnormalities in rats with SCI. Furthermore, this extract also stimulated neuronal restoration in the injured spinal cord by inhibiting the inflammatory processes and upregulating the expression of neuroprotective factors (VEGF and Bcl-xL) [92].

Wang et al. showed that ginseng treatment significantly downregulated oxidative stress on spinal injury in rats by enhancing antioxidant proteins and decreasing inflammatory proteins and proinflammatory cytokines [93].

Moreover, different doses of *P. ginseng* showed a significant improvement in locomotor function after spinal injury in rats. *P. ginseng* treatments decreased the expression of COX-2 and iNOS at the lesion site and the cavity area. These results suggest that *P. ginseng* may improve the recovery of motor function after SCI [91].

#### 4.1.6. Panax Notoginsenoside

Unlike many other herbal medicines with a highly variable range of applications, *Panax notoginseng*, which is classified as a warm, sweet, slightly bitter, and nontoxic in Chinese medicine, has protective actions against cardiovascular diseases and diabetes [94, 95]. Moreover, many pharmacological activities of *P. notoginseng*, such as antioxidant, anti-inflammatory, hypoglycemic, antihyperlipidemic, anticoagulation, neuroprotective, and hepatoprotective effects, as well as antitumor and estrogen-like activities, have been reviewed [96].

Over 200 chemical constituents, including saponins, flavonoids, phytosterols, saccharides, polysaccharides, amino acids, fatty acids, dencichine, cyclopeptides, volatile oils, aliphatic acetylene hydrocarbons, and trace elements, have been isolated from *P. notoginseng* [97].

Panax notoginsenoside (PNS) is the principal active ingredient extracted from *P. notoginseng*, which main components are ginsenoside Rb1 (29.86%), Rg1 (20.46%), Rd (7.96%), Re (6.83%), and notoginsenoside R1 (2.74%) [98]. PNS shows many beneficial effects, including anti-inflammation, antiedema, antioxidation, and antiapoptosis [96, 99], as well as neuroprotection in animal models of cerebral ischemia/reperfusion injury [100].

Compelling neuroprotective effects of PNS were demonstrated in a spinal cord ischemia-reperfusion injury model. SCI was induced in rats previously treated with PNS, in which the BBB scores significantly increased, as well as the number of neurons and a restored neuronal morphology observed by a histopathological examination. Furthermore, PNS decreased cytokine levels, as well as the expression of AQP-4 after the injury, suggesting an antiedema effect. An antiapoptotic mechanism of PNS was also verified since the treatment reduced the expression of Fas and FasL and inhibited injury-induced apoptosis [101].

#### 4.1.7. Ginkgo biloba Extract 761 (EGb-761)

Ginkgo biloba (Ginkgoaceae) is an ancient Chinese tree which has been cultivated and held sacred for its health-promoting properties [102]. EGb-761 is a patented extract from the leaves of the Ginkgo biloba tree composed of flavonoids (24%), ginkgolide (3.1%), bilobalides (2.9%), and organic acids (5-10%), particularly a ginkgo glycoside [103].

As one of the EGb-761 major constituents, ginkgolide B can improve hemorrhage, edema, necrosis, and inflammatory cell infiltrates in the injured spinal cord [39].

EGb-761 decreases blood viscosity, thereby increasing microcirculation [104], and modifies neurotransmission [105] and neuroplasticity as well [106]. Ginkgo biloba extracts have been used for the treatment of diseases related to the CNS, including brain injury, neurodegenerative disorders, and degenerative dementia [107–109].

EGb-761 also enhances cognition, reduces the detrimental effects of ischemia [110], shows neuroprotective effects, and enhances neurogenesis after ischemic stroke [111].

EGb-761 possesses antioxidant and free radical-scavenging activities [112, 113]. In neurons treated with hydrogen peroxide, EGb-761 reduced oxidative stress and increased the viability of neurons [114, 115]. EGb-761 inhibits xanthine oxidase activity [116], reduces the production of superoxide anion, and inhibits SOD in human postmortem brain tissue [117]. By using electron spin resonance spectrometry, it was demonstrated that EGb-761 is a potent superoxide anion scavenger with SOD activity [118]. Also, EGb-761 can scavenge peroxyl radicals [119].

In spinal cord ischemic injury, EGb-761 protected spinal cord neurons *in vivo*, as well as hydrogen peroxide-induced spinal cord neuronal death *in vitro* [120]. During the acute phase after SCI, EGb-761 administration significantly reduced secondary injury-induced tissue necrosis and cell apoptosis and improved functional performance in rats [121].

EGb-761 performs its neuroprotective effects through scavenging free radicals, lowering oxidative stress, reducing neural damage, and preventing platelet aggregation, as well as its anti-inflammatory properties [122–124].

EGb-761 protects against ischemic SCI via their antioxidant effects in a rat model [125]. Furthermore, EGb-761 decreases SOD downregulation and significantly reduces MDA levels in spinal cord ischemia/reperfusion in rabbits [126, 127]. In another study, EGb-761 was not able to demonstrate a uniform effect on the biochemical markers of spinal cord ischemia/reperfusion in rats. However, histopathologic data appear to show a protective effect of EGb-761 on the spinal cord tissue [128].

Research has demonstrated that iNOS expression is upregulated after SCI. In contrast, EGb761 can suppress iNOS expression and prevent neuronal death in SCI rats: in the treated group, the area of cavities was smaller, and the demyelinated zones were limited at and around the site of the SCI in comparison to the control group [129].

In acute spinal cord contusion in rats, cell apoptosis increased until day 14 after the injury. Furthermore, seven days after the injury, the number of apoptotic cells significantly decreased in the EGb761-treated group [121].

#### 4.1.8. Hyperforin

*Hypericum perforatum*, also known as St. John's wort, hypericum, or millepertuis, is a member of the family *Hypericaceae*. Native from Europe, western Asia, and northern Africa, this herbaceous perennial plant currently can be found worldwide. The crude drug, known as *Hyperici herba*, is collected from the upper part of the aerial parts of the plant before or during the flowering period [130–132].

*H. perforatum* has demonstrated neuroprotective activities. Neuroprotection can be achieved by a direct action on one or several mechanisms, such as an antiapoptotic effect, or indirectly, through antioxidant properties. Chemically, structure-activity relationships suggest that a sugar side chain of flavonoids might be essential for neuroprotective activities [133] and multiple hydroxyl groups confer substantial antioxidant properties [134].

The active component of *H. perforatum* is hyperforin [135, 136], which reduces the overload of [Ca^2+^] through NMDA receptor modulation in neurons [136, 137]. *H. perforatum* standardized extract protects against enzymatic and nonenzymatic lipid peroxidation of rat brain, inhibiting NADPH-dependent lipid peroxidation and attenuating nonenzymatic Fe^2+^/ascorbate-dependent lipid peroxidation in cerebral cortex mitochondria [138].

After SCI in rats, *H. perforatum* showed a reduction in oxidative stress, apoptosis, and intracellular Ca^2+^ influx values through the regulation of the TRPM2 (transient receptor potential melastatin 2) and TRPV1 (transient receptor potential vanilloid 1) channels in dorsal root ganglion (DRG) neurons. Additionally, *H. perforatum* induced protective effects on lipid peroxidation and decreased GSH-Px values in the DRG neurons [139].

#### 4.1.9. Rosmarinic Acid and Carnosol

Rosmarinic acid (RA) is a polyphenol found in the *Lamiaceae* family, abundantly present in rosemary, sage, lemon balm, and thyme. RA is a natural antioxidant with free radical scavenging and potential biological effects against oxidative stress and inflammation [140–142].

Shang et al. showed that RA treatment significantly decreased oxidative stress and enhanced the antioxidant status in post-SCI rats. Treatment with RA regulated redox homeostasis and oxidative stress markers, such as ROS, lipid peroxides, and sulfhydryl and carbonyl groups in proteins. RA treatment also caused the upregulation in Nrf-2 levels with the concomitant increase in antioxidant enzymes, such as SOD, CAT, GPx, GST, and GSH, and exerted anti-inflammatory effects through the downregulation of NF-*κ*B and proinflammatory cytokine levels (IL-6, IL-1*β*, TNF-*α*, and MCP-1) after SCI [143].

Carnosol, an orthodiphenolic diterpene with excellent antioxidant potential, is also found in rosemary. Wang et al. showed the protective role of carnosol against SCI-induced oxidative stress and inflammation through modulating NF-*κ*B, COX-2, and Nrf-2 levels in Wistar rats. After the significant increase in ROS generation, oxidant levels, lipid peroxide content, protein carbonyl and sulfhydryl levels, and the reduction of the antioxidant status generated by induced SCI, carnosol treatment regulated inflammation key proteins and redox status through the significant downregulation of NF-*κ*B and COX-2 levels and the upregulation of p-AKT and Nrf-2 expression [144].

#### 4.1.10. Silymarin and Silybin

Silymarin (SM) is a mixture of flavonoids extracted from *Silybum marianum* (milk thistle) plant, including flavonolignans (silybin A and silybin B, isosilybin A, isosilybin B, silychristin, and silydianin), as well as fatty acids and polyphenols [145]. SM can contribute to antioxidant defenses as a scavenger of free radicals and by inhibiting specific free radical production enzymes. It also maintains an appropriate redox status by activating some enzymes and nonenzymatic antioxidants via transcription factors, including Nrf2 and NF-*κ*B. Furthermore, SM activates the synthesis of protective molecules, such as thioredoxin (Trx), heat shock proteins, and sirtuins [146].

SM and silybin inhibited cell proliferation in cultures of glial cells in a concentration-dependent manner, as well as mixed cortical and spinal neuronal/glial cells against peroxide toxicity, and protected spinal cord neuronal/glial, glial and microglial cell cultures from LPS stimulation or peroxide toxicity. SM and silybin attenuated peroxide-induced ROS formation, with SM being more effective than silybin. Moreover, intrathecal administration of SM immediately after SCI effectively improved hindlimb locomotor behavior in the rats. These findings showed that SM and silybin exhibit general neuroprotective actions in the CNS [147].

Silybin elicits neuroprotection by the inhibition of peroxide-induced ROS through neuroinflammation and activation of glial cells, by modulating NF-*κ*B or protein kinase C (PKC), as well as apoptosis, through inhibiting p53 and caspase-9, among other signaling pathways [147, 148].

### 4.2. Extracts from Roots or Bulbs

#### 4.2.1. Plumbagin

Plumbagin, an analog of vitamin K3 isolated from the root of *Plumbago zeylanica L*, activates the Nrf2/ARE pathway resulting in the upregulation of target genes and increased resistance to oxidative and metabolic insults of neurons in culture and to ischemic stroke *in vivo* [149]. In a rat model, post-SCI treatment with plumbagin reduced SCI-induced oxidative stress and proinflammatory mediators [150]. SCI decreased the antioxidant levels of both nonenzymatic (GSH) and enzymatic antioxidants (NQO1, GST, GPx, SOD, and CAT) in sham rats. However, a significant increase in the antioxidant pool was observed in SCI rats treated with plumbagin.

Moreover, it is well-known that Nrf2 activates the antioxidant machinery of cells [151]. Interestingly, plumbagin showed a significant upregulation of Nrf-2 in SCI, which suggests an essential role of plumbagin in cytoprotection as a potent Nrf-2 inducer [150].

#### 4.2.2. Tetrandrine

Tetrandrine (TET), a bis-benzylisoquinoline alkaloid extracted from the roots of the Chinese medicinal herb *Stephania tetrandrae* S Moore, is a potential therapeutic candidate against cancer [152, 153], inflammation [154], and brain ischemia/reperfusion injury [155].

TET is a calcium channel blocker that can protect the liver, heart, small bowel, and brain from ischemia/reperfusion injury by inhibiting damaging factors, such as lipid peroxidation, generation of reactive oxygen species, production of cytokines and inflammatory mediators, neutrophil recruitment, and platelet aggregation [156].

Bao et al. studied the protective effect of TET on rat spinal cord astrocytes with oxygen-glucose-serum deprivation/reoxygenation-induced injury [157]. As expected, this intentional insult which mimics hypoxic/ischemic conditions *in vivo* caused considerable oxidative stress: an increase in ROS and MDA content, as well as a decreased SOD activity. Also, it increased the expression of proapoptotic Bax and caspase-3 proteins, as well as the reduction of the antiapoptotic protein Bcl-2 [158]. The results of TET as a pretreatment to oxygen-glucose-serum deprivation/reoxygenation injury showed a dose-dependent suppression of Akt phosphorylation and NF-*κ*B activity and inhibition of the elevated caspase-3 activity. Additionally, TNF-*α*, IL-1*β*, and IL-6 accumulation induced by hypoxic/ischemic conditions were diminished. Overall, these results show a protective effect of TET against hypoxic/ischemic injury in spinal cord astrocytes through the PI3K/AKT/NF-*κ*B signaling pathway attributable to its antioxidant and anti-inflammatory properties [157].

#### 4.2.3. Puerarin

Puerarin, a natural isoflavone, is the main constituent of *Radix Puerariae lobata*. In SCI, puerarin has shown neuroprotective effects by improving motor function, mainly in ischemia-reperfusion [159–161] as well as in traumatic injury models [162]. Some mechanisms of neuroprotection have been described. As SCI causes the elevation of glutamate levels and mGluRs mRNA expression, which lead to neuronal injury, it has been shown that puerarin administration decreases both the excessive delivery of glutamate and the activation of mGluRs [160]. Also, puerarin upregulates the expression of GAP43, promoting the regeneration of nerve fibers [163]. Another mechanism of protection by puerarin is the inhibition of Cdk5 and p25. Cdk25 causes neuronal death and often is accompanied by the accumulation of p35 and p25, which in turn activates Cdk5, inducing feedback for the stimulus of the injury [161].

Moreover, puerarin can attenuate histological damage, decrease neuron death, and inhibit glial cell activation. These effects can be promoted by increasing the activity of the PI3K/Akt signaling pathway, which is involved in axonal outgrowth and the promotion of antioxidant and antiapoptosis effects [162].

Puerarin diminishes neuroinflammation [162, 164] by decreasing the activity of NF-*κ*B and proinflammatory cytokines, such as IL-6, IL-1*β*, and TNF-*α* [164]. Regarding apoptosis, puerarin reduces ROS by increasing thioredoxin- (TRX-) 1/TRX-2 mRNA levels, which are known to regulate apoptosis by modulating the redox ratio of the cell [159].

#### 4.2.4. Allicin

*Allium sativum* (garlic) is a common and tasty ingredient found all over the world, which also has been used for medicinal purposes. In ancient Chinese and Indian medicine, it was used to treat several conditions, including leprosy, infections, and snake bites. Throughout history, garlic has been used to treat cardiovascular diseases and reduce high blood glucose concentration, blood pressure, and cholesterol levels. More recently, its antitumor, anti-inflammatory, antifungal, and antimicrobial effects have been studied [165, 166].

Garlic contains many substances, from which allicin is the principal chemical component responsible for its biological activity [167].

Allicin is formed during the chopping, crushing, or chewing of garlic cloves through a chemical interaction between alliin, a sulfur-containing amino acid, and the enzyme alliinase [168] and has been reported to prevent arteriosclerosis, stenocardia, cerebral infarction, arrhythmia, and hydrargyria, as well as to enhance the immune system and reduce oxidation [166, 169].

Liu et al. found that allicin treatment for glutamate-induced oxidative stress in spinal cord neurons significantly reduced LDH release, loss of cell viability, and apoptotic neuronal death. Allicin effects were associated with reduced oxidative stress, as evidenced by decreased ROS generation, reduced lipid peroxidation, and preservation of antioxidant enzyme activities. Also, allicin diminished the expression of iNOS and significantly increased the expression of heat shock protein 70 (HSP70) at both mRNA and protein levels. Knockdown of HSP70 by specific targeted small interfering RNA (siRNA) not only mitigated allicin-induced protective activity but also partially nullified its effects on the regulation of iNOS [167]. Furthermore, when the beneficial effects of allicin on SCI in mice were investigated, the results showed that allicin significantly increased BBB scores, which was associated with the inhibition of oxidative stress and inflammatory responses. It was also demonstrated that allicin increased the levels of HSP70, increased the phosphorylation of Akt, and reduced the iNOS protein expression levels. Additionally, treatment with allicin significantly reduced ROS and enhanced NADH levels [170]. Liu et al. and Wang and Ren results agreed and collectively demonstrated that the beneficial effects of allicin are mediated by the HSP70/Akt/iNOS pathway and recognized its potential use for SCI treatment [167, 170].

#### 4.2.5. Curcumin

Curcumin (1,7-bis(4-hydroxy-3-methoxyphenyl)-1,6-heptadiene-3,5-dione) is a nonsteroidal, naturally occurring compound found in an Indian spice commonly used as a dietary pigment known as turmeric. Curcumin exhibits a variety of pharmacologic effects, including anti-inflammatory, anticarcinogenic, anti-infectious, antioxidant, and hypocholesterolemic activities. Diets containing curcumin have shown to stimulate NGF, BDNF, GDNF, and PDGF levels *in vivo* [171, 172]. Curcumin also enhances neurogenesis and synaptogenesis and improves cognition in rats, as well as in clinical trials for different neurodegenerative diseases [173], probably through promoting these neurotrophic factors.

After spinal cord hemisection, curcumin treatment provides neuroprotection against SCI-induced disability in rats by the attenuation of neuron loss, prevention of neuronal apoptosis, and decreasing astrocyte activation. Curcumin can attenuate astrocyte reactivation *in vitro* by downregulating GFAP expression, which may improve neuronal survival [174].

### 4.3. Extracts from Fruits or Derivatives

#### 4.3.1. Quercetin

Many fruits and vegetables contain quercetin (3,3′,4′,5,7-pentahydroxyflavone), a common flavonol [175]. Together with flavones, anthocyanidins, and other compounds, flavonols belong to the class of flavonoids, which in turn represent a major class of polyphenols [176].

Like other polyphenols, quercetin is a scavenger of ROS and reactive nitrogen species such as NO and ONOO [177]. As well as its metabolites, quercetin acts by modulating the antioxidant defense mechanisms in the cell [178, 179].

The beneficial effects of quercetin on cardiovascular diseases, cancer, infections, inflammatory processes, gastrointestinal tract function, and diabetes have been reported [177, 180, 181]. Moreover, quercetin can exert neuroprotection [182] and antagonize oxidative stress when orally administered *in vivo*. At a dose of 0.5-50 mg/kg, quercetin protected rodents from oxidative stress and neurotoxicity induced by different insults [183, 184]. Also, quercetin reduces the immunoreactivity of degenerating neurons [185] and promotes neuronal recovery through the inhibition of inflammatory responses [186].

In a recent study, it was observed that quercetin treatment following acute SCI in rats promoted electrophysiological and locomotor recovery, reduced cavity formation, and contributed to astrocyte activation and axonal regeneration. Additionally, quercetin increased the expression of the brain-derived neurotrophic factor (BDNF), although it reduced p-JNK2 and p-STAT3 expression [187, 188]. It has been reported that BDNF activates tropomyosin-related kinase B (Trk-B) through several downstream signaling pathways, such as AKT, CaMK, and Ras/Raf/MEK/ERK, leading to cell survival, growth, and neuroplasticity [189], while the JAK2/STAT3 pathway depends on the binding of erythropoietin (EPO) to a receptor that results in the dimerization of JAK2. This dimerization leads to STAT3 and STAT5 phosphorylation and the formation of stable homodimers and heterodimers, which subsequently induce the transcription of genes that regulate cell proliferation and survival [190]. Consequently, it was proposed that quercetin effects possibly worked through BDNF and JAK2/STAT3 signaling pathways [188].

#### 4.3.2. Tocotrienols

Tocotrienols, isomers of vitamin E, are found in some cereal and vegetable derivatives, such as palm oil, rice bran oil, coconut oil, barley germ, wheat germ, and annatto. Other sources of tocotrienols include grape seed oil, oat, hazelnuts, maize, olive oil, Buckthorn berry, rye, flaxseed oil, poppy seed oil, and sunflower oil [191, 192].

Tocotrienols exhibit strong neuroprotective, antioxidant, and anticancer effects and cholesterol-lowering properties, which are not observed in tocopherols [193]. Due to a better distribution in the lipid layers of the cell membrane, experimental evidence has found that tocotrienols function as better antioxidants and free radical scavengers when compared to tocopherols [194].

In a rat model, tocotrienol protected against SCI by reducing oxidative stress and inflammation and inhibiting iNOS protein expression and activity, as well as plasma NO production. Also, treatment with tocotrienols suppressed TGF-*β*, collagen type IV, and fibronectin protein expression levels. Furthermore, the BBB scores in rats treated with 120 mg/kg/day tocotrienol were significantly higher when compared with the group administered with MPS sodium succinate [38].

#### 4.3.3. Resveratrol

Resveratrol is a naturally occurring stilbene class of polyphenol produced in the skin of many edible plants as a response to fungal infection [195].

The resveratrol-mediated decrease in neuronal MDA levels is often associated with increased activation of antioxidant enzymes such as SOD [196] and antioxidant compounds such as glutathione (GSH) [197].

The antioxidant enzyme HO-1 is implicated particularly as a significant effector of resveratrol-mediated neuroprotection after postischemic reperfusion [198, 199]. Furthermore, resveratrol treatment induces HO-1 expression in cultured mouse cortical neurons [200].

Resveratrol ameliorates kainate-induced excitotoxicity [201]. Subsequently, resveratrol has been shown to improve histopathological and behavioral outcomes after various types of acute CNS injuries, including stroke [202–204], traumatic brain injury [205, 206], subarachnoid hemorrhage [207], and SCI [208–210].

In moderate damage to the spinal cord, Liu et al. showed that injured animals treated with resveratrol showed a significant increase in BBB scores. Furthermore, after resveratrol administration, the histopathological analysis showed a restored neuronal morphology and increased the number of neurons. Concerning the antioxidation effects of resveratrol, the treatment overturned the decreased SOD activity and the increased MDA levels caused by SCI, which suggests an antioxidation effect after the injury. Resveratrol treatment also showed an anti-inflammation effect after SCI by inhibiting immunoreactivity and the expression of inflammatory cytokines, such as IL-1*β*, IL-10, TNF-*α*, and myeloperoxidase (MPO). Finally, an antiapoptosis role of resveratrol was observed by the inhibition of injury-induced apoptosis and the modulation of the expression of apoptosis-related genes Bax, Bcl-2, and caspase-3 [210].

### 4.4. Other Extracts

#### 4.4.1. *Tithonia diversifolia* Extracts

As mentioned above, Chinese and European traditional medicine is vast. However, it is not the only option to offer proposals for future acute SCI treatments. An example of African traditional medicine is *Tithonia diversifolia*, which proved to possess anti-inflammatory properties to treat diabetes mellitus, diarrhea, fever, hematomas, hepatitis, hepatomas, malaria, and wounds [211, 212]. *T. diversifolia* is a bushy perennial weed that can be found in Nigerian fields, as well as in wastelands and roadsides in Taiwan.

Phytochemical investigations have identified the existence of some bioactive compounds, such as chromene, flavone, cadinene, germacrene, eudesmane, and rearranged eudesmane derivatives in *T. diversifolia* [213–215].

Juang et al. obtained *T. diversifolia* ethanolic extracts (TDE) and used it to treat rats with T5 static compression as a model of SCI. First, these researchers noticed that SCI increased the water apparent diffusion coefficient (ADC)—a measurement of the diffusion of water molecules within the central nervous system—after six hours. A low value of ADC indicates that the nerve fiber tracts are well organized, while a high value means that the tracts are damaged and disorganized. TDE treatment slightly decreased the ADC level after one week in the SCI model. Therefore, it was proposed that TDE protects cells against hydrogen peroxide or radical scavenging-induced toxicity through an antioxidant mechanism, which might be responsible for cell neuroprotection [216].

#### 4.4.2. Danshen Extracts

Danshen (*Salvia miltiorrhiza Bunge*) is a traditional Chinese herb used for the treatment of heart, liver, and skin diseases, among others. Danshen crude extracts (DCE) attenuate edema and bleeding. Furthermore, DCE treatment improved spinal cord microcirculation, as well as motor function by elevating GDNF mRNA expression in the gray matter of acute SCI in rats [217]. Moreover, DCE increased the expression of the antiapoptotic gene Bcl-2, decreased the number of TUNEL-positive cells, decreased MDA levels, and increased the expression of superoxide dismutase as well [218], which demonstrated that DCE treatment decreased apoptosis and showed beneficial effects over oxidative stress in SCI.

Several chemical components, such as salvianolic acid B (Sal B), 3,4-dihydroxyphenyl lactic acid (DLA), and tanshinone IIA (TIIA), are obtained from Danshen extracts.

Salvianolic acid B (Sal B) is commonly used for the prevention and treatment of cardiovascular disease and shows neuroprotective effects in animal models.

Sal B improves motor function [219, 220]. One probable mechanism of Sal B is through the oligodendrocyte precursor cell differentiation due to the increase of the myelin sheath and the number of regenerating axons. These observations indicate that Sal B can protect axons and the myelin sheath [221].

Furthermore, Sal B has shown anti-inflammatory effects by attenuating the upregulation of TNF-*α* and NF-*κ*B [219]. Moreover, Sal B activates proapoptotic mediators as caspase-3 [220, 221].

Interestingly, Sal B regulates the blood-spinal cord barrier (BSCB) permeability and can reduce spinal edema [220]. In this case, Sal B upregulated the expression of ZO-1 and occludin mediated by HO-1, and p-caveolin was significantly decreased as well [219, 220]. Additionally, Sal A induced the expression of miR-101, which regulates BSCB integrity via the miR-101/Cul3/Nrf2/HO-1 signaling pathway [220].

DLA is obtained by water extraction and improves motor function and tissue damage in SCI. Moreover, it shows its effects on the inflammatory response by reducing I*κ*B-*α* degradation and the nuclear translocation of NF-*κ*B p65 subunit, as well as polymorphonuclear cell infiltration and IL-6 production [222].

TIIA is one of the principal components of Danshen, which has shown anti-inflammatory and antiapoptotic effects on several diseases: activates blood circulation and exerts neuroprotective effects. In SCI, TIIA improves motor function and reduces tissue injury [223–225]. A possible mechanism for TIIA is the low activation of astrocytes and upregulated expression of Nestin, NeuN, and NF200, indicating that TIIA can promote cell differentiation [225]. The anti-inflammatory mechanisms are carried out by the inhibition of the activation of NF-*κ*B, MAPK, and JNK pathways and the downregulation of proinflammatory cytokines TNF-*α*, IL-1*β*, IL-6, and iNOS. TIIA decreases neutrophil and monocyte infiltration by decreasing the myeloperoxidase activity [223, 226]. Also, the anti-inflammation induced by TIIA has shown positive responses to neuropathic pain [226].

TIIA reduces apoptosis by decreasing caspase-3 activation and upregulating Bcl-2 [223, 224, 227]. Finally, TIIA increases the expression of heat-shock protein 70 and inhibits Bax expression [224] and shows effects on redox state imbalance and antiedema [223, 227].

## 5. Discussion

Different cellular and molecular targets are currently under investigation to improve the outcome after SCI. However, no strategies that effectively improve the secondary damage underlying SCI are currently approved by the FDA. Due to the complexity of SCI—in which secondary posttraumatic mechanisms produce neuronal degeneration associated with increased oxidative stress and inflammation—and the lack of therapeutic options, further investigation of other treatments becomes necessary to improve the quality of life of patients with this lesion.

This review describes several compounds derived from plants, vegetables, and fruits that have been tested in SCI models, in which they exhibit antioxidant, anti-inflammatory, and antiapoptotic therapeutic potential. These properties come from compounds such as the asiatic acid, obtained from *Centella asiatica*, and plumbagin, an analog of vitamin K3 isolated from *Plumbago zeylanica* L, which increase the levels of antioxidant enzymes. Ligustilide is a bioactive ingredient that reduces oxidative stress and inflammation. Tetrandrine, which is extracted from the root of *Stephania tetrandrae*, is a compound with neuroprotective effects through blocking calcium canals. Consequently, it reduces the molecules associated with oxidative stress damage. *Danshen* extracts decreased apoptosis and oxidative stress and improved motor function in acute SCI. Ginsenosides extracted from *P. ginseng* promote neuronal restoration, inhibit inflammatory processes, and downregulate oxidative stress.

Puerarin, the main constituent of *Radix Puerariae lobata*, and tetramethylpyrazine, extracted from *Ligusticum wallichii Franchat*, showed neuroprotective, as well as antioxidant and antiapoptosis effects, and also reduced neuroinflammation against SCI.

EGb-761, an extract from *Ginkgo biloba*, improved functional performance after SCI, due to its antioxidant, free radical scavenging, and antiapoptosis properties.

Some polyphenols, including quercetin, rosmarinic acid, silymarin, epigallocatechin-3-gallate, and resveratrol, are ROS and reactive nitrogen species scavengers. Therefore, they contribute to regulating oxidative stress. Lastly, curcumin is a nonsteroidal compound with a variety of pharmacological effects.

Some limitations of this review were the lack of information about the use of other natural compounds from Mexican and Latin American countries—which are known to possess an impactful millennial tradition in the use of medicinal plants—to treat SCI. However, this opens up the possibility of future research for new American natural compounds with antioxidant properties, which could be used as a potential treatment for SCI as well. Due to the heterogeneity between the beginning, the duration, and the routes of administration of the treatments, it is impossible to compare their efficiency and strength. Therefore, another limitation could be the lack of standard procedures that allow the comparison of the effectiveness of these compounds.

Although many natural compounds have been used in SCI, little is known about their effectiveness as natural antioxidants, the mechanisms through which these compounds exert their antioxidant activities or their ability to cross the BBB in preclinical models.

As a conclusion, 21 compounds commonly used in SCI models with beneficial properties were described in this review. These compounds are potential therapeutic candidates with neuroprotective effects such as reducing the levels of ROS and diminishing oxidative stress.

Even though these compounds have been tested in animal models with promising results, no clinical studies have been conducted in humans. Therefore, it is crucial to design some strategies to study the effects of these natural compounds in patients with SCI, given that most of these plants are available worldwide at a much lower cost than some synthetic drugs used for SCI therapy.

## Figures and Tables

**Table 1:** Antioxidant mechanisms and effects of natural compounds or extracts on SCI models.

Compound or extract	Plant species	Origin	Effects on SCI models	Antioxidant mechanisms	References
*From leaves*					
Asiatic acid (AA)	*Centella asiatica*	China	Improves scores in locomotion tests Anti-inflammatory and antioxidant properties	Increases SOD activity and GSH content	[41–44]
Ligustilide (LIG)	*Radix angelicae sinensis* *Ligusticum chuanxiong*	China	Neuroprotective, cardiovascular, anti-inflammatory, and antioxidant effects	Scavenger, significantly suppresses iROS and iNOS expression Inhibits PLC*γ* and increases PI levels	[49, 50]
Tetramethylpyrazine (TMP)	*Ligusticum wallichii Franchat*	China	Neuroprotective effects Anti-inflammatory properties	Increases GSH levels and SOD activity, activates the Akt/Nrf2/HO-1 pathway	[55–60]
Epigallocatechin-3-gallate (EGCG)	*Camellia sinensis* (green tea)	China Japan Korea	Improves motor function Relieves neuropathic pain Anti-inflammatory and antioxidant properties	Increases GR and MDA levels and reduces myeloperoxidase and iNOS activity	[67, 68, 70, 77]
Ginsenosides	*Panax ginseng* C.A. Meyer	Asia	Neuroprotective effects Antioxidant properties	Enhances the Nrf2/Ho-1 pathway and antioxidant proteins, decreases COX-2 and iNOS expression	[80–85]
Panax notoginsenoside (PNS)	*Panax notoginseng* (sachi ginseng)	China Burma Nepal	Anti-inflammatory, antioxidant, neuroprotective, and antiapoptotic effects	Activation of Nrf2 and upregulation of downstream antioxidant systems, including HO-1 and GSTP1	[93, 96, 99, 100]
Ginkgo biloba extract 761 (EGb-761)	*Ginkgo biloba*	China	Neuroprotective effects Anti-inflammatory and antioxidant properties	Scavenger, inhibits xanthine oxidase and iNOS activity, reduces superoxide anion with SOD activity	[112–129]
Hyperforin	*Hypericum perforatum* (St. John's wort)	Europe Asia Africa	Antioxidant properties Antiapoptotic effects	Decreases G-Px values, inhibits NADPH-dependent lipid peroxidation, and attenuates nonenzymatic Fe^2+^/ascorbate-dependent lipid peroxidation	[133–139]
Rosmarinic acid (RA)	*Lamiaceae* family (rosemary, sage, lemon balm, and thyme)	India	Anti-inflammatory and antioxidant properties	Scavenger, upregulation in Nrf-2 levels with the concomitant increase in antioxidant enzymes (SOD, CAT, G-Px, GST, and GSH)	[140–143]
Carnosol	*Rosmarinus officinalis* L	Worldwide	Anti-inflammatory and antioxidant properties	Upregulates p-AKT and Nrf-2 expression	[144]
Silymarin (SM), silybin	*Silybum marianum* (milk thistle)	Mediterranean diet North Africa	Induce effective functional, recovery Antioxidant properties	Scavenger, inhibits ROS production enzymes, activates antioxidant enzymes via transcription factors (Nrf2 and NF-*κ*B)	[146, 147]
*From roots or bulbs*					
Plumbagin	*Plumbago zeylanica* L	India and Ceylon	Anti-inflammatory and antioxidant properties	Activates the Nrf2/ARE pathway by which antioxidant enzymes are increased	[150, 151]
Tetrandrine (TET)	*Stephania tetrandrae* S	China	Protection against hypoxic/ischemic injury	Scavenger, blocks iNOS and COX-2 expression	[154–157]
Puerarin	*Radix Puerariae lobata*	China Worldwide	Anti-inflammation and antiapoptotic properties	Increases TRX-1/TRX-2 mRNA levels and the activity of the PI3K/Akt signaling pathway	[159–164]
Allicin	*Allium sativum* (garlic)	Asia	Improves scores in locomotion tests Inhibition of oxidative stress	Reduces iNOS protein expression levels, enhances NADH levels	[167–170]
Curcumin	*Curcuma longa* (turmeric)	India China Southeast Asia	Antioxidant and anti-inflammatoryproperties	Scavenger, restores mitochondrial membrane potential, upregulates Cu-Zn SOD, improves GSH levels, inhibits iNOS expression by suppressing NF-*κ*B signaling pathway, and activates transcription factors AP-1 and Nrf2	[171–173]
*From fruits*					
Quercetin	Apples, berries, onions, broccoli, red grapes, and caper	America Europe Asia Africa	Exerts neuroprotection Promotes locomotor function recovery Axonal regeneration	Scavenger of ROS and reactive nitrogen species	[177–184]
Tocotrienols	Certain cereals, palm oil, rice bran oil, coconut oil, barley germ, wheat germ and annatto, grape seed oil, oat, hazelnuts, maize, olive oil	America Europe Asia Africa Oceania	Improves scores in locomotion tests Anti-inflammatory and antioxidant properties	Scavenger, inhibits iNOS protein expression and activity	[38, 193, 194]
Resveratrol	*Polygonum cuspidatum*, red wine, red grape skins, berries, blueberries, and peanuts	China Japan Korea America Europe Africa	Improved scores in locomotion tests Increased number of neurons Antioxidant properties Antiapoptotic effects	Increases activation of antioxidant enzymes such as SOD and antioxidant compounds such as GSH, induces HO-1 expression	[196–199]
*Other extracts*					
Sesquiterpenoids, flavonoids, phenols	*Tithonia diversifolia*	Africa Asia	Neuroprotection Antioxidant properties	Scavenger	[216]
Salvianolic acid B (Sal B), 3,4-dihydroxyphenyl lactic acid (DLA), tanshinone IIA (TIIA)	*Salvia miltiorrhiza Bunge* (Danshen)	China	Neuroprotection Antioxidant properties Antiapoptotic effects BSCB integrity regulation	Increases SOD expression, upregulates the expression of HO-1, decreases myeloperoxidase activity	[220–227]

SOD: superoxide dismutase; GSH: glutathione; iROS: intracellular reactive oxygen species; iNOS: inducible nitric oxide synthase; PLC*γ*: phospholipase C*γ*; PI: phosphoinositol; GR: glutathione reductase; MDA: malondialdehyde; HO-1: heme oxygenase-1; GSTP1: glutathione S-transferase P1; G-Px: glutathione peroxidase; CAT: catalase; GST: glutathione transferase; TRX: thioredoxin; BSCB: blood-spinal cord barrier.
